# Lasting differential gene expression of circulating CD8 T cells in chronic HCV infection with cirrhosis identifies a role for Hedgehog signaling in cellular hyperfunction

**DOI:** 10.3389/fimmu.2024.1375485

**Published:** 2024-06-03

**Authors:** Jiafeng Li, Agatha Vranjkovic, Daniel Read, Sean P. Delaney, William L. Stanford, Curtis L. Cooper, Angela M. Crawley

**Affiliations:** ^1^ Chronic Disease Program, Ottawa Hospital Research Institute, Ottawa, ON, Canada; ^2^ Department of Biochemistry, Microbiology and Immunology, University of Ottawa, Ottawa, ON, Canada; ^3^ Centre for Infection, Immunity and Inflammation, University of Ottawa, Ottawa, ON, Canada; ^4^ Regenerative Medicine Program, Ottawa Hospital Research Institute, Ottawa, ON, Canada; ^5^ Department of Cellular and Molecular Medicine, University of Ottawa, Ottawa, ON, Canada; ^6^ Ottawa Institute of Systems Biology, University of Ottawa, Ottawa, ON, Canada; ^7^ Division of Infectious Diseases, The Ottawa Hospital, Ottawa, ON, Canada; ^8^ School of Epidemiology and Public Health, University of Ottawa, Ottawa, ON, Canada; ^9^ Clinical Epidemiology Program, Ottawa Hospital Research Institute, Ottawa, ON, Canada; ^10^ Department of Biology and Institute of Biochemistry, Carleton University, Ottawa, ON, Canada

**Keywords:** hepatitis C virus, liver cirrhosis, advanced liver fibrosis, CD8 T cells, T cell dysfunction, gene expression, Hedgehog signaling, direct-acting antivirals (DAA)

## Abstract

**Background:**

The impact of chronic hepatic infection on antigen non-specific immune cells in circulation remains poorly understood. We reported lasting global hyperfunction of peripheral CD8 T cells in HCV-infected individuals with cirrhosis. Whether gene expression patterns in bulk CD8 T cells are associated with the severity of liver fibrosis in HCV infection is not known.

**Methods:**

RNA sequencing of blood CD8 T cells from treatment naïve, HCV-infected individuals with minimal (Metavir F0-1 ≤ 7.0 kPa) or advanced fibrosis or cirrhosis (F4 ≥ 12.5 kPa), before and after direct-acting antiviral therapy, was performed. CD8 T cell function was assessed by flow cytometry.

**Results:**

In CD8 T cells from pre-DAA patients with advanced compared to minimal fibrosis, Gene Ontology analysis and Gene Set Enrichment Analysis identified differential gene expression related to cellular function and metabolism, including upregulated Hedgehog (Hh) signaling, IFN-α, -γ, TGF-β response genes, apoptosis, apical surface pathways, phospholipase signaling, phosphatidyl-choline/inositol activity, and second-messenger-mediated signaling. In contrast, genes in pathways associated with nuclear processes, RNA transport, cytoskeletal dynamics, cMyc/E2F regulation, oxidative phosphorylation, and mTOR signaling, were reduced. Hh signaling pathway was the top featured gene set upregulated in cirrhotics, wherein hallmark genes *GLI1* and *PTCH1* ranked highly. Inhibition of Smo-dependent Hh signaling ablated the expression of IFN-γ and perforin in stimulated CD8 T cells from chronic HCV-infected patients with advanced compared to minimal fibrosis. CD8 T cell gene expression profiles post-DAA remained clustered with pre-DAA profiles and disparately between advanced and minimal fibrosis, suggesting a persistent perturbation of gene expression long after viral clearance.

**Conclusions:**

This analysis of bulk CD8 T cell gene expression in chronic HCV infection suggests considerable reprogramming of the CD8 T cell pool in the cirrhotic state. Increased Hh signaling in cirrhosis may contribute to generalized CD8 T cell hyperfunction observed in chronic HCV infection. Understanding the lasting nature of immune cell dysfunction may help mitigate remaining clinical challenges after HCV clearance and more generally, improve long term outcomes for individuals with severe liver disease.

## 1 Introduction

The complications accompanying advanced liver disease remain a major global health burden. Chronic viral hepatitis is a leading factor contributing to this burden of liver disease (1, 2). Antiviral treatments for hepatic viral infections such as hepatitis C (HCV) and B (HBV) have emerged to eliminate or control viremia. However, the long-term outcome for those with cirrhosis remains poor for many affected individuals (3). As liver damage progresses to advanced liver fibrosis (AF) and cirrhosis, there is an increased risk for progression to end-stage liver disease, portal hypertension, esophageal varices, hepatic encephalopathy, susceptibility to infections, and hepatocellular carcinoma (HCC) (4–6). While several studies demonstrate that direct-acting antivirals (DAA) reduce HCC risk in HCV-infected individuals (7–10), there still remains appreciable incidence following completion of curative treatment (5). In addition to the associated metabolic disease in cirrhosis, a variety of immune dysfunctions emerge as liver disease progresses, affecting both innate and adaptive immune responses (11). While innate immune dysfunctions have been well described in cirrhosis (12–14), the contribution of adaptive immune defects to the health outcomes of cirrhosis has not been fully examined.

Chronic HCV infection disrupts many innate and adaptive immune cells, including cytotoxic CD8 T cells (15–20). In the acute stage of HCV infection, weak and transient responses of HCV-specific CD8 T cells predict chronicity (21, 22). In chronic infection, detection of impaired HCV-specific CD8 T cells prior to IFN-α and ribavirin antiviral therapy predicts chronic infection upon reinfection (23), as these cells remain dysfunctional (17, 24), suggesting irreversible damage to immune cells. It remains unclear if viral cure with DAA parallels with restored immune functions (25, 26). In addition, HCV infection has an extensive, antigen agnostic effect on CD8 T cells, as markers of exhaustion are widely observed on bulk CD8 T cells in the blood, spleen and liver (27–30). A study has observed exaggerated proliferation, cytokine secretion and degranulation by *in vitro-*stimulated cytomegalovirus or Epstein-Barr virus (CMV/EBV)-specific CD8 T cells in HCV-infected individuals and this was retained after DAA therapy (31). However, the effects of liver fibrosis severity on the acquisition and possible long-term retention of T cell dysfunction have not been determined. Attempts to restore normal function *in vitro* in isolated HCV/CMV/EBV-specific CD8 T cells from HCV-infected individuals with AF have encountered challenges (32). This suggests that the immune system is profoundly affected in HCV infection according to the degree of liver fibrosis.

We and others have observed extensive impairment of the entire CD8 T cell compartment in the blood and liver in HCV infection (27–30, 33, 34), wherein we specifically associated decreased CD8 T cell survival with AF (33). We then showed for the first time an overactive bulk CD8 T cell function profile in HCV-infected individuals with cirrhosis compared to those with minimal fibrosis (MF) (34). This was done alongside our complimentary clinical study in which a cohort of DAA-treated HCV^+^ patients with cirrhosis achieved a sustained virological response, SVR (i.e. undetectable HCV RNA by 12 weeks after treatment cessation) yet failed to reverse liver fibrosis by 24-weeks after viral clearance (35). We hypothesized that liver fibrosis severity is strongly associated with immune dysfunction. Consistent with this theory, we found that after DAA therapy, bulk CD8 T cell responses were not restored to levels comparable to healthy individuals in patients with cirrhosis (34).

Characterizing the underlying mechanisms of bulk CD8 T cell dysfunction in AF is of clinical importance given the role of CD8 T cells in response to infection and cancer surveillance. In this study, we identify several candidate genes and pathways that may contribute to this hyperfunction and inform future mechanistic investigations, and highlight an important impact on Hedgehog (Hh) signaling. Hh signaling is widely recognized as an important component of embryonic development and tissue regeneration (36, 37). The signaling cascade is initiated by extracellular Hh ligands (homologs Sonic, Desert and Indian) binding to receptor Patched-1 (Ptch-1) or Patched-2 (Ptch-2), and results in, through Smoothened (Smo), downstream activation of Gli transcription factors (Gli1, Gli2, and Gli3). Non-canonical Gli-independent Hh signaling also plays a prominent role in Ca^2+^ signaling and cytoskeletal rearrangement (38). More recently, it has been shown that exposure to Sonic Hh ligand produced by adult thymic epithelial cells is essential to drive differentiation and proliferation of thymocytes during transition through the double-negative stages of development (39). Hh signaling was also found to support γδ T cell maturation (40). In mice, Hh signaling was also shown to be involved in the formation of the immunological synapse of CD8 T cells (41). This study is thus complemented with data showing a dependence on Hh signaling in CD8 T cell function and its contribution to immune cell hyperfunction in chronic HCV with AF.

## 2 Materials and methods

### 2.1 Study subjects

Study subjects (**Table 1**) were treatment-naïve, chronically infected with HCV (>6 months HCV RNA^+^). All DAA-treated individuals studied achieved SVR unless otherwise specified. This research was conducted in accordance with the guidelines established by the Ottawa Health Science Network Research Ethics Board. Study participants were consented, and blood samples were collected by staff at The Ottawa Hospital Clinical Investigations Unit.

**Table 1:** Summary of demographics and clinical information of study groups.

Parameter	Samples used in RNA-seq^*^		Samples used in PCR and T cell functional analyses^*^		
	HCV^+^ AF (F3-F4)^a^	HCV^+^ MF (F0-1)^a^	HCV^+^ AF (F3-F4)^a^	HCV^+^ MF (F0-1)^a^	Uninfected
Sample size	4	4	4	4	4
Sex	3 M, 1 F	1 M, 3 F	3 M, 1 F	3 M, 1 F	2 M, 2 F
Mean age ± SD (Rage)	58.5 ± 12.4 (46−75)	48.5 ± 5.0 (41−51)	53.8 ± 7.1 (46−62)	52.5 ± 15.2 (33−65)	47.5 ± 10.6 (35−59)
Ethnicity	Caucasian (3), S-E Asian (1)	Caucasian (4)	First Nations (2), Caucasian (1), Unknown (1)	Caucasian (3), Unknown (1)	Caucasian (2), South-American (1), Unknown (1)
Mean fibrosis score^b^ (kPa) ± SD	23.9 ± 13.6	3.9 ± 1.7	16.2 ± 3.4	5.3 ± 1.1	Not applicable

a AF = Advanced fibrosis, METAVIR F3-F4 ≥ 12.5 kPa; MF = Minimal fibrosis, METAVIR F0-1 ≤ 7.0 kPa.

b Liver stiffness in kilopascal (kPa) measured by Fibroscan transient elastography.

^*^Samples reported in RNA-seq are different individuals than samples reported in qPCR and T cell function.

### 2.2 CD8 T cell isolation and culture

PBMCs of study participants were isolated by Lymphoprep™ density gradient centrifugation (StemCell™ Technologies, Canada), and cryopreserved in heat-inactivated fetal-bovine serum (HI-FBS, ThermoFisher Gibco™, USA) + 10% (v/v) dimethyl sulfoxide (Sigma-Aldrich, USA) at 1×10^7^ viable cells/ml. At the time of use, cryopreserved PBMCs were thawed and rested for 16h in RPMI 1640 (ThermoFisher Gibco™) + 10% (v/v) HI-FBS + 100 U/ml penicillin-streptomycin (pen-strep, ThermoFisher Gibco™) at 37°C, 5% CO_2_. CD8 T cells were then isolated by magnetic bead positive selection (StemCell™ Technologies). Isolated CD8 T cells were cultured at 1×10^6^ cells/ml in complete RPMI (RPMI 1640 + 20% (v/v) HI-FBS + 100 U/ml pen-strep) at 37°C, 5% CO_2_ for all experiments.

### 2.3 RNA-sequencing of CD8 T cells

Isolated CD8 T cells were stimulated in culture with 0.5 µg/ml phytohemaglutanin-L (PHA-L, Sigma-Aldrich) for 18h. Following stimulation, total RNA was isolated using TRIzol™ Reagent (ThermoFisher Invitrogen™) following manufacturer’s protocol. Total RNA yields were determined by spectrophotometer (ThermoFisher NanoDrop™ ND-1000) analysis of the A260/A280 ratio. To enable performance quality assessment during sequencing, spike-in control RNA (ThermoFisher Ambion™) was added to all samples at a 1:100 dilution following manufacturer’s protocol.

RNA-sequencing was carried out by the Donnelly Sequencing Centre (DSC) in Toronto, Canada. Briefly, the purity and integrity (threshold RNA Integrity Number >8) of isolated total RNA was determined by microfluidic spectrophotometry on the 2100 Bioanalyzer (Agilent Technologies, USA). RNA-seq libraries were generated via mRNA isolation from total RNA by polyA-positive selection using the TruSeq™ RNA/DNA Library Preparation Kit (Illumina, USA) prior to sequencing using the NextSeq™ 550 system (Illumina).

### 2.4 RNA-seq data analysis

Bioinformatical analysis of RNA-seq data was carried out in collaboration with the Ottawa Bioinformatics Core Facility (OHRI and University of Ottawa, Ottawa, Canada) using the R programming language. Read mapping was performed using the *Salmon* tool (42) and quality control was subsequently performed using the *HISAT2* tool (43). Fold-change analysis was then performed using the *DESeq2* tool (44), applying for each gene a cutoff of ≥5 detectable reads in ≥2 samples for retention, which removes non-expressed and non-detectable transcripts, based on the *tximport* transcripts library (45). Hierarchical clustering was calculated using all detectable transcripts. The top 500 most variably expressed genes across the samples were used to generate the principal component analysis plot. Statistically significant differentially expressed genes between study groups were identified using the *aleglm* method (46) prior to GSEA (47, 48) and GO enrichment analysis (49–51). GO classifications with ≥3 enriched genes per term were kept as enriched classifications.

### 2.5 RT-qPCR of Hh signaling genes in CD8 T cells

Isolated CD8 T cells were stimulated in culture with anti-CD3/CD28 antibodies. Briefly, high-binding 96-well plates were coated with 5 μg/ml anti-CD3 (Clone UCHT1, BD Pharmingen™, USA) in PBS (ThermoFisher Gibco™) for 1h at 37°C, 5% CO_2_ prior to seeding cells for culture (conditions described above). Soluble anti-CD28 (Clone CD28.2, BD Pharmingen™) was then added to the cells at 2 μg/ml, and cells were cultured for 16h. Following stimulation, total RNA was isolated using the RNeasy Plus Micro kit (QIEGEN, Netherlands) following manufacturer’s protocol. Total RNA yields were determined by spectrophotometry using NanoDrop™ One (ThermoFisher) analysis of the A260/A280 ratio, and RNA purity and integrity was determined by capillary electrophoresis on the 5200 Fragment Analyzer (Agilent Technologies) at StemCore Laboratories (OHRI). cDNA was generated using the iScript™ cDNA Synthesis Kit (Bio-Rad, USA) following manufacturer’s protocol, and gene expression was assessed by qPCR using the SYBR Green reporter (Bio-Rad) on the CFX Connect (Bio-Rad) using the following PrimePCR™ (Bio-Rad) primer assays (*GENE*, Assay ID): *PTCH1*, qHsaCED0001809; *GLI1*, qHsaCID0011958; *TBP* (housekeeping gene), qHsaCID0007122. Fold-change of gene expression between study groups was calculated using the 2^-ΔCt^ method normalized to *TBP*.

### 2.6 Inhibition of Hh signaling in CD8 T cells

Isolated CD8 T cells were stimulated in culture with anti-CD3/CD28 antibodies as described above for 48h, with or without 50 μM Smo inhibitor cyclopamine (StemCell™ Technologies). The expression of IFN-γ and perforin in CD8 T cell subsets were analyzed by spectral flow cytometry on the Aurora (Cytek Bioscience, USA) using the following conjugates and markers (Clone, Fluorophore; BioLegend, USA): Viability dye (Zombie Aqua™), CD8 (RPA-T8, BV785), CCR7 (G043H7, APC-Cy7), CD45RA (HI100, BV650), IFN-γ (4S.B3, PE-Cy7), Perforin (B-D48, APC). Data was analyzed with FlowJo™ v.10 software (BD) and statistical analysis was performed with Prism v.10 software (Dotmatics GraphPad™, USA).

## 3 Results

### 3.1 Differential gene expression in bulk CD8 T cells in HCV infection with advanced or minimal liver fibrosis is associated with cellular metabolism, cell structure, and motility

**Figure 1:**
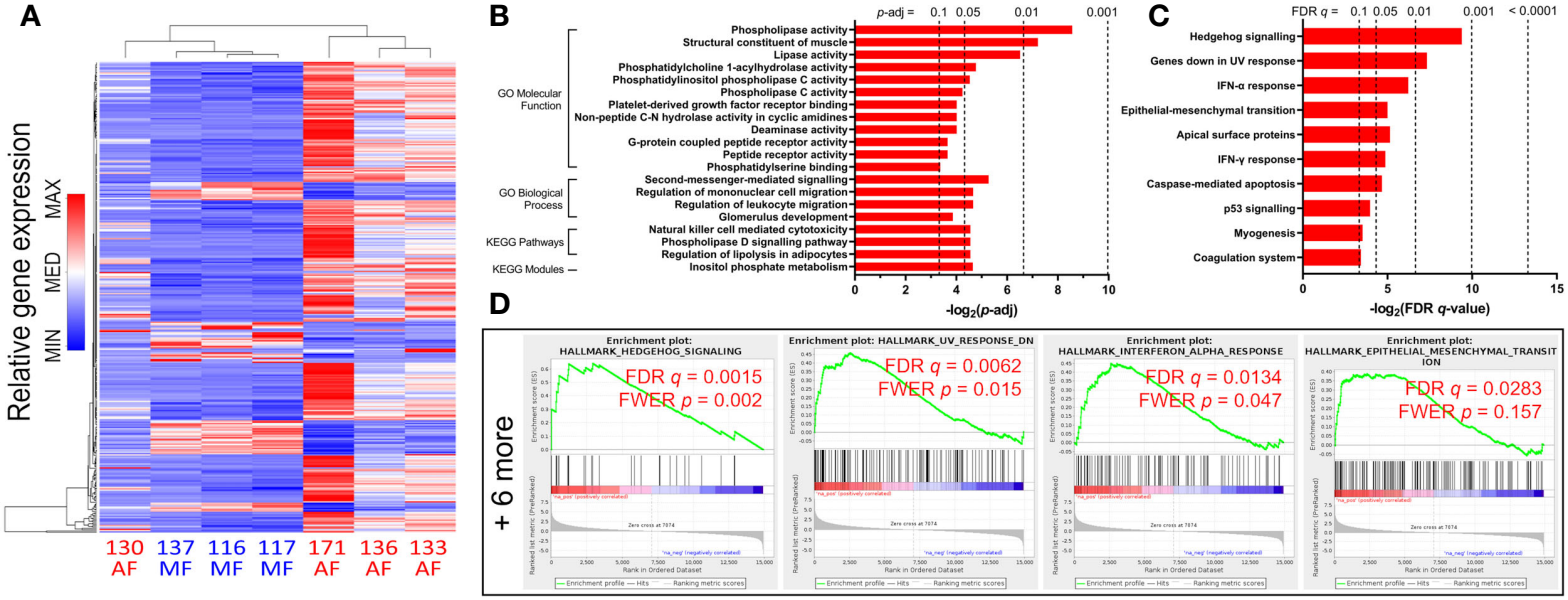
Gene expression changes and functional enrichment of upregulated pathways in untreated HCV-infected individuals with advanced fibrosis (AF) compared to minimal fibrosis (MF). **(A)** Complete-linkage heatmap clustering of all 362 genes that are differentially expressed between AF and MF patients show clear distinct gene expression profile differences. **(B)** Gene Ontology (GO) analyses of upregulated genes in CD8 T cells from AF patients compared to MF identifies multiple classifications related to inflammatory responses and metabolic regulation. **(C)** Gene Set Enrichment Analysis (GSEA) enriched 10 gene sets upregulated in AF, notably Hh signaling and inflammatory responses. **(D)** GSEA enrichment plots of the top four upregulated gene sets (Hh signaling, genes downregulated in UV response, IFN-α response, and epithelial-mesenchymal transition) in AF compared to MF.

Analyses of bulk CD8 T cells in HCV-infected subjects based on liver fibrosis severity are sparse. Our previous work demonstrated CD8 T cell hyperfunction in blood cells from HCV-infected individuals with AF prior to DAA therapy when compared to those with MF (34). The gene expression profiles of 8 HCV-positive, treatment-naïve individuals (**Table 1**) were thus examined. Four exhibited MF (METAVIR F0-1, liver stiffness ≤ 7.0 kPa) and four had AF (F4 ≥ 12.5 kPa). All received DAA therapy and cleared the virus (i.e. achieved SVR). We isolated bulk CD8 T cells from PBMC samples collected from these study groups pre-DAA, followed by 16h stimulation with 5μg/ml phytohaemagglutinin (PHA) and RNA-sequencing. In total, 24,058 detectable genes were retained for analysis out of a database of 58,294. A total of 362 genes were significantly differentially expressed (*p*-adj<0.05), of which 288 genes were upregulated and 74 were downregulated. Complete-linkage clustering of these genes separated CD8 T cell gene expression profiles of AF individuals (patients 133, 136, 171) from MF (patients 116, 117, 124, 137), with patient 130 as the exception (**Figure 1A**).

To determine which cellular functions are modulated by these gene expression patterns, we performed functional enrichment analysis of the lists of differentially expressed genes in HCV-infected individuals with cirrhosis or MF before DAA treatment. The enrichment analysis searches curated databases of functional categories and highlighted gene sets that may be statistically over-represented in the dataset (47, 49, 50). The adjusted *p*-value cutoff of 0.1 was used to identify enriched groups. Each term or gene set hit in these analyses contains a set of genes with correlating expression patterns, annotated by biological pathway or function.

An analysis for Gene Ontology (GO) Molecular Function (MF) and Biological Processes (BP) classifications was performed. GO MF terms associated with upregulated genes in the cirrhosis group compared to the MF group include phospholipase activity (*p* = 0.003), lipase activity (*p* = 0.011), and phospholipase C activity (*p* = 0.053) (**Figure 1B**). GO BP terms associated with upregulated genes include second-messenger-mediated signaling (*p* = 0.026), and regulation of leukocyte migration (*p* = 0.040) (**Figure 1B**). An enrichment analysis in Kyoto Encyclopedia of Genes and Genomes (KEGG) pathways and modules was also performed. Notable KEGG hits enriched for upregulated gens in cirrhosis include NK cell-mediated cytotoxicity (*p* = 0.043), and phospholipase D signaling (*p* = 0.043) (**Figure 1B**). Additionally, gene set enrichment analysis (GSEA) was conducted on significantly differentially expressed genes in cirrhosis. In total, 10 GSEA upregulated gene sets were enriched (FDR *q* ≤ 0.1) in CD8 T cells from cirrhotic individuals compared to MF, including Hh signaling (specifically genes for Patched-1 and Gli1, *PTCH1* and *GLI1*, with core enrichment ranking of 1 and 4 respectively), IFN-α and -γ responses, and apoptosis (**Figures 1C, D**).

**Figure 2:**
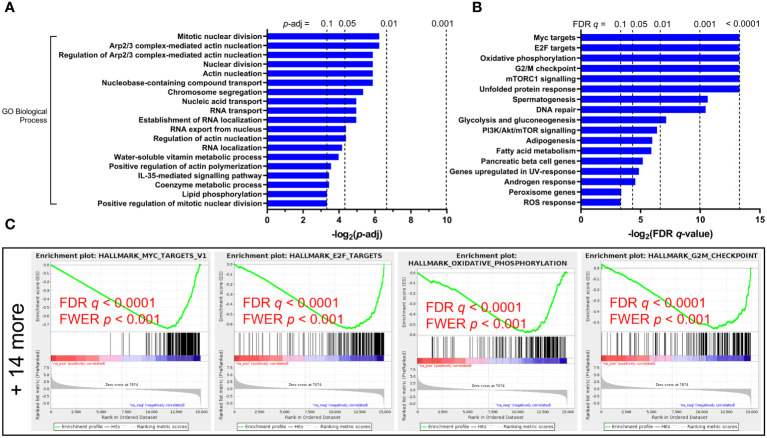
Functional enrichment of downregulated pathways in untreated HCV-infected individuals with advanced fibrosis (AF) compared to minimal fibrosis (MF). **(A)** Gene Ontology (GO) Biological Processes analysis of downregulated genes in AF identifies multiple classifications related to cytoskeletal regulation and nucleic acid transport regulation. **(B)** Gene Set Enrichment Analysis (GSEA) enriched 18 gene sets downregulated in AF, many of which relate to nucleic acid transport and metabolic regulation. **(C)** GSEA enrichment plots of the top four downregulated gene sets (Myc targets, E2F targets, oxidative phosphorylation, and G2/M checkpoint) in AF compared to MF.

In genes downregulated in the cirrhosis group, no GO MF terms nor KEGG pathways or modules were enriched, while GO BP terms enriched include nuclear division (p = 0.013), actin nucleation (p = 0.017), as well as RNA transport (p = 0.032) and localization (p = 0.056) (**Figure 2A**). GSEA hits in downregulated genes include Myc and E2F targets (54 and 130 genes respectively), oxidative phosphorylation (122 genes), G2/M checkpoint (112 genes), mTORC1 signaling (93 genes), and DNA repair (68 genes) (**Figures 2B, C**).

Overall, these analyses indicate that the gene expression differences in CD8 T cells from chronic HCV patients with cirrhosis spread across many vital signaling pathways and cellular processes. These differences hinge mostly upon functions associated with cellular metabolism and cell growth, T cell activation and inflammatory responses, RNA transport, as well as cytoskeletal control and cellular migration.

### 3.2 Inhibition of canonical Hedgehog signaling ablates CD8 T cell hyperfunction in AF

Given the importance of Hh signaling in mediating T cell development and cytotoxic functions (39, 41, 52), and its enrichment as leading GSEA hit (**Figure 1C**), we confirmed differential RNA expression in CD8 T cells in disparate liver fibrosis severities in HCV infection by qPCR analysis of *PTCH1*, *SMO*, and *GLI1* mRNA. We observed increased *PTCH1* mRNA in cells from HCV^+^ individuals associating with fibrosis severity and no observable increases in *SMO* expression, as expected based on sequencing results, although we were unable to observe increases in *GLI1* mRNA expression (**Supplementary Figure 1**).

**Figure 3:**
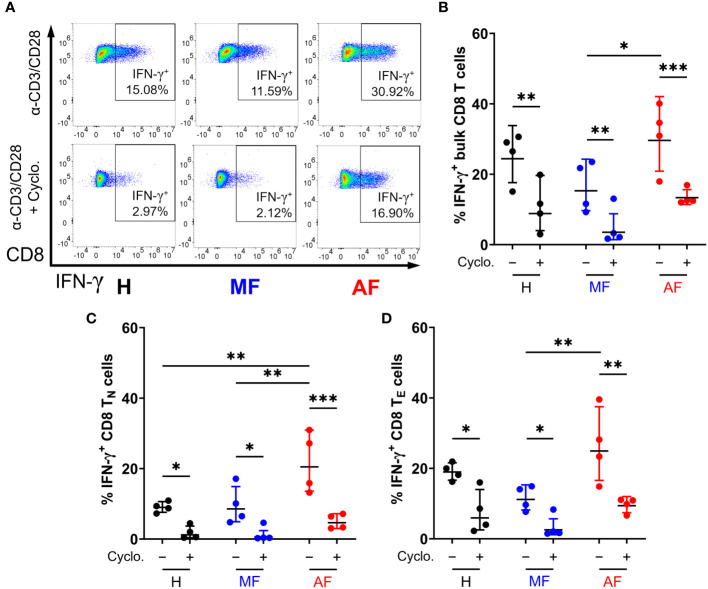
Inhibition of Smo-dependent Hedgehog (Hh) signaling ablates IFN-γ expression in CD8 T cells. **(A)** Representative dot plots of IFN-γ expression in bulk CD8 T cells from untreated HCV-infected individuals with advanced fibrosis (AF), minimal fibrosis (MF), or healthy controls (H), stimulated with anti-CD3/CD28 antibodies with or without 50 µM of cyclopamine (Cyclo). Smo inhibition with cyclopamine during stimulation ablated IFN-γ expression in **(B)** bulk, **(C)** naïve (T_N_), and **(D)** effector (T_E_) CD8 T cell subsets, where hyperfunction was observed. Multiple comparisons are analyzed by 2-way ANOVA with Šídák’s post-test, *p ≤ 0.05, **p ≤ 0.01, ***p ≤ 0.001.

**Figure 4:**
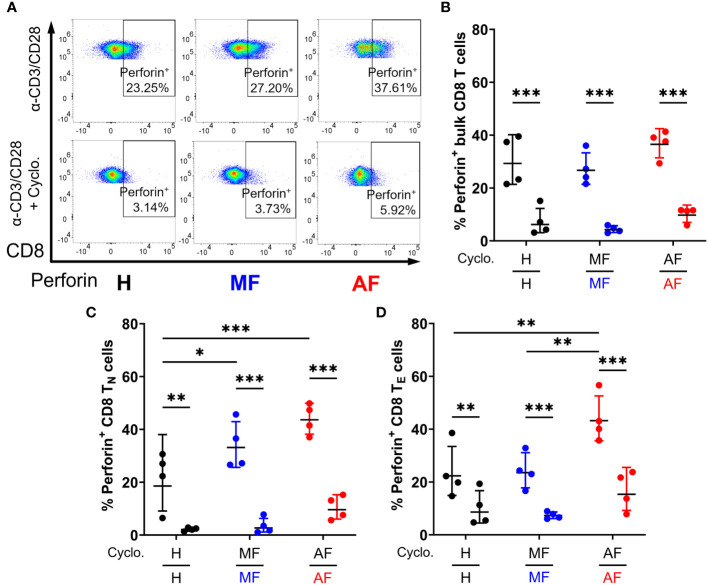
Inhibition of Smo-dependent Hedgehog (Hh) signaling ablates perforin expression in CD8 T cells. **(A)** Representative dot plots of perforin expression in bulk CD8 T cells from untreated HCV-infected individuals with advanced fibrosis (AF), minimal fibrosis (MF), or healthy controls (H), stimulated with anti-CD3/CD28 antibodies with or without cyclopamine. Smo inhibition with cyclopamine during stimulation ablated perforin expression in **(B)** bulk, **(C)** naïve (T_N_), and **(D)** effector (T_E_) CD8 T cell subsets, where hyperfunction was observed. Multiple comparisons are analyzed by 2-way ANOVA with Šídák’s post-test, *p ≤ 0.05, **p ≤ 0.01, ***p ≤ 0.001.

We next examined whether Hh signaling contributes to IFN-γ and perforin expression in CD8 T cells. Smo is a central mediator in Hh signaling, and Smo-dependency is a major target of interest (53–55). Peripheral CD8 T cells isolated from HCV patients and healthy individuals (**Table 1**) were stimulated for 48h with anti-CD3 and anti-CD28 antibodies, with or without cyclopamine, an FDA-approved chemical inhibitor of Smo, prior to IFN-γ and perforin expression analysis by flow cytometry. CD8 T cell subsets were defined as follows: naïve (T_N_, CCR7^+^CD45RA^+^), effector (T_E_, CCR7^−^CD45RA^+^), effector memory (T_EM_, CCR7^−^CD45RA^−^), and central memory (T_CM_, CCR7^+^CD45RA^−^), as outlined in **Supplementary Figure 2**. Mirroring previous reports, CD8 T cells from cirrhotic individuals exhibit hyperfunction through increased IFN-γ and perforin expression across various cell subsets, notably in naïve cells and effector cells (**Figures 3**, **4**, AF *vs* MF *vs* H without cyclopamine). Cyclopamine treatment (50 μM) of CD8 T cells alongside anti-CD3/CD28 stimulation ablated IFN-γ expression in bulk CD8 T cells regardless of liver disease severity (**Figures 3A, B**), in a dose-dependent manner (**Supplementary Figure 3**). This dependence on Smo-mediated Hh signaling was also observed in naïve and effector cells in AF where hyperfunction was observed (**Figures 3C, D**). Perforin expression by CD8 T cells treated with cyclopamine was also ablated in bulk cells (**Figures 4A, B**), again identified in naïve and effector cells (**Figures 4C, D**) where hyperfunction was observed. Taken together, this suggests that canonical Hh signaling plays an important role in IFN-γ and perforin expression during the CD8 T cell response and may play a role in bulk CD8 T cell hyperfunction in AF in HCV^+^ individuals.

### 3.3 CD8 T cell gene expression differences persist after DAA-mediated viral clearance

The persistence of CD8 T cell dysfunction post-DAA therapy is well reported (34, 56–59). However, association with fibrosis severity remains understudied. Our previous study showing systemic CD8 T cell hyperfunction in chronic HCV cirrhosis also showed persistence of hyperfunction 24 weeks post-SVR (34). Therefore, gene expression profiles of CD8 T cells isolated from post-DAA chronic HCV patients with cirrhosis was assessed and compared to minimal fibrosis, in parallel to pre-DAA profiling described above.

**Figure 5:**
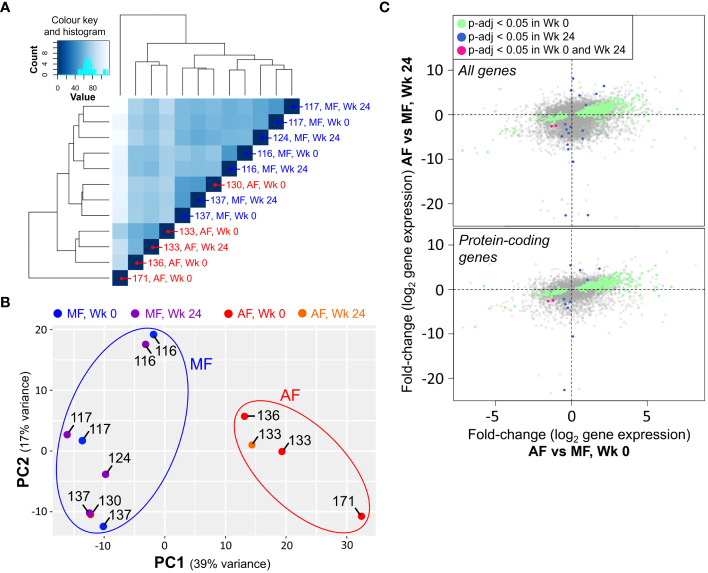
CD8 T cell gene expression patterns in chronic HCV persists after direct-acting antiviral (DAA) treatment. **(A)** Hierarchical clustering of RNA-seq data from isolated and stimulated CD8 T cells based on all transcripts shows clear separation between gene expression patterns of cells from AF and MF patients, independent of treatment stage (Wk 0: Week 0, pre-DAA; Wk 24: Week 24, 12 weeks post-SVR). **(B)** PCA applied to the top 500 most variable genes separates AF and MF patients into distinct clusters independent of treatment stage. **(C)** Plotting gene expression fold-change differences at Wk 0 compared to Wk 24 shows an association across all differentially expressed (*p*<0.05) genes (top), as well as well as differentially expressed protein-coding genes (bottom).

Hierarchical clustering based on normalized gene expression counts across all transcripts identified a clear clustering between samples from individuals with AF compared to MF (**Figure 5A**). Principal component analysis (PCA) applied to the top 500 most variable genes in this dataset also revealed clear differences in gene expression in patients with AF before treatment compared to that of individuals with MF (**Figure 5B**). PCA broadly separated the MF patients (patients #116, 117 and 137) from AF patients (patients #133, 136 and 171), with one exception (patient #130), consistent with heatmap clustering of only pre-DAA samples (**Figure 1A**). Where applicable, the CD8 T cell gene expression profiles of treatment-paired patient samples (pre- vs. post-DAA) remained constant in the three individuals with MF (patients #116, 117 and 137) as well as the single DAA-treated individual with high fibrosis (patient #133). Fold-change differences based on fibrosis severity before treatment moderately correlated with severity-based differences after treatment (**Figure 5C**), indicating an altered gene expression profile that persists after DAA treatment.

Taken together, this suggests that CD8 T cell gene expression is greatly affected by the severity of liver fibrosis in chronic HCV infection, and that this difference is particularly noticeable before DAA intervention, but appears to remain after DAA-mediated viral clearance. Due to the relatively small available post-DAA sample size at the time of gene expression data acquisition, we specifically report below on untreated HCV-infected individuals evaluated based on liver fibrosis severity (AF vs MF).

## 4 Discussion

We report that bulk circulating CD8 T cells from HCV-infected individuals exhibit differential gene expression patterns based on liver fibrosis severity after *in vitro* stimulation. Altered expression of genes associated with CD8 T cell function, survival, cellular metabolism, and cytoskeletal dynamics was identified through RNA-sequencing and subsequent bioinformatical discovery (**Figures 1**, **2**). While the RNA-sequencing data was generated by PHA stimulation of isolated CD8 T cells, which is an established method to assess T cell function (60), we ensured that the identified pathways were investigated using the more physiologically relevant stimulation with anti-CD3/CD28.

In chronic HCV infection, HCV-specific CD8 T cell responses are largely characterized by an immune exhaustion phenotype (15, 56, 61, 62). However, it is increasingly recognized that inflammatory cytokines facilitate T cell activation in the context of viral infection, enabling cells to circumvent antigen-dependency through an innate-like response (63). Upregulated IFN-γ response genes in cells from patients with cirrhosis correlates with our previous finding of elevated proportions of IFN-γ^+^ cells in HCV infection (34), although in a different context with this report showing an increase in genes downstream of IFN-γ signaling rather than IFN-γ genes themselves. Such parallels may be expected, as IFN-γ autocrine and paracrine signaling in mouse CD8 T cells has been shown to enhance motility and cytotoxicity, promoting T-bet and granzyme B expression (64, 65). Our data also identified an increased IFN-α responsiveness in CD8 T cells of HCV^+^ patients with cirrhosis compared to MF. It has been reported that IFN-α stimulation of PBMCs resulted in a phenotypic shift in CMV-/EBV-specific CD8 T cells from healthy individuals to that resembling cells in chronic HCV infection, with upregulated PD-1, Tim-3 and 2B4 expression (31). IFN-α plays a role in supporting T cell receptor engagement and co-stimulation, similar to IL-12, by enhancing CD8 T cell differentiation and function (66). IFN-α has also been shown to directly increase IFN-γ and granzyme production by naïve or antigen-experienced CD8 T cells (67, 68). Such inflammatory environment of chronic infection also leads to increased CD8 T cell death (69, 70), which was corroborated by our GSEA identification of apoptosis genes and ongoing studies (Crawley unpublished). Together, these RNA-seq data suggests the potential involvement of non-antigen-specific activation and function of CD8 T cells in HCV infection with AF.

Across several GSEA and GO enrichment hits, changes in genes involved in cytoskeletal regulation further suggest dysregulation of cellular structure in CD8 T cells from HCV^+^ individuals with AF. These include identified upregulation in leukocyte migration gene sets, as well as downregulation in four gene sets associated with the regulation and activation of actin nucleation (*p* < 0.05, **Figure 2**), with a fifth as a trend toward the downregulation of genes associated with the positive regulation of actin polymerization. The ability of CD8 T cells to enact receptor-mediated intracellular signaling and release cytotoxic molecules hinges on tight actin filament regulation (71, 72). Actin and microtubule reorganization, as well as inositol phosphate metabolism which were enriched in our RNA-seq data, play a central role in mediating the targeted synaptic release of cytolytic granules centrosomes (72). Given these gene expression changes and the strong upregulation of Hh signaling in GSEA, which acts on the actin organization of CD8 T cells (41), intracellular structural changes may be an important driver of CD8 T cell hyperfunction in advanced liver disease and impact their roles in responses to infection or cancer surveillance. Inositol triphosphate signaling, as well as phospholipase signaling, also play an important role in T cell receptor signaling in development and function (73).

In addition, non-canonical Hh signaling may be vital in T cell function. Smo is known to induce phosphorylation of ATP-promoting AMPK (74, 75), which is involved in T cell function (76), and Gli activity is regulated by cAMP-responsive PKA, among others (38, 77). Hh signaling is also involved in mediating T_H2_ and T_reg_ polarization (78–80), controlling T_H17_ polarization via Smo-dependent AMPK signaling (75), mediating CD8 T cell cytotoxic actions (41), and may enhance T cell co-stimulation (39, 81). Reduced Hh signaling in exhausted CD4 T cells in Tb is thought to indicate weaker inflammatory response and immune activity (82). Increased plasma Hh ligands from hepatocytes, is observed in chronic HBV and HCV (83, 84). Given these roles of Hh signaling in metabolic regulation and T cell responses, it is likely that its dysregulation in chronic HCV is a leading factor in CD8 T cell hyperfunction.

The degree of cellular heterogeneity in bulk circulating CD8 T cells, based on surface phenotypes alone, should be considered in the interpretation of such bulk RNA sequencing. In patients with multiple sclerosis, heterogeneity of gene expression has been documented in naïve CD4 T cells that suggest bias in gene expression potential can exist prior to antigen encounter (85). Even cytotoxic CD8 T cells exhibit inherent heterogeneity, with IFN-γ-expressing cells being most responsible for cytolytic activities with the production of TNF-α, granzymes, perforin and chemokines associated with antimicrobial activity (e.g. CCL5), whereas IL-2-producing cells modulate immune response with IL-4, -3 and -11 cytokine production (86). The increased expression of CCL5 suggesting potential co-expression with lytic molecules such as perforin is in agreement with our previous finding that CD8 T cells from cirrhotic patients express elevated levels of perforin (34). Continuous systemic stimuli in chronic infection, such as persistently high levels of serum Hh ligands, could also influence cellular heterogeneity as well (83, 87, 88). Additional studies are required to identify the CD8 T cell subsets responsible for generalized immune cell hyperfunction in cirrhosis.

Although our dataset is small, the gene expression profiles between AF and MF were sufficiently different to suggest that gene expression patterns may not be readily reverted after curative antiviral treatment, in-line with our previous observations of long-lasting hyperfunction (34). This persistent dysfunction suggests that the observed CD8 T cell hyperfunction in HCV cirrhosis is due to cellular defects that are not readily reversed by the resolution of HCV viremia. Consequently, the persistence of dysfunctional CD8 T cells likely contributes to remaining adverse clinical outcomes in HCV-infected individuals with cirrhosis, despite long after achieving SVR. Generalized CD8 T cell dysfunction in cirrhosis may reduce immunocompetence and lead to increased risk of community-acquired infections such as pneumonia (89, 90) and poor responses to routine vaccinations in HCV-infected individuals (91–95). Landmark studies in murine chronic LCMV infection show how CD8 T cell dysfunction associates with several weakened aspects of the immune response (96, 97). HCV vaccines currently under development seek to emulate T cell responses required for protection against HCV, including reinfection (98). However, it is thought that lasting CD8 T cell dysfunction after HCV cure will result in a failure to generate effective HCV vaccine responses in vulnerable populations (99) and potentially contribute to the development of HCC and poor responses to immunotherapy.

To date, this is the first study to our knowledge to have probed generalized CD8 T cell gene expression patterns in chronic HCV infection in the context of liver disease severity. Evaluating the immune function in the context of chronic HCV-derived liver disease severity can be difficult due to confounding inter-individual immunological factors, such as minor infections/inflammation, and in the case of this study, small sample sizes. This has been reflected in many data sets underrepresenting participants with cirrhosis. Furthermore, the ability to conduct longitudinal studies to evaluate responses before and long after DAA therapy is difficult given the commonly high loss-to-follow-up rates of chronic HCV studies (100, 101). It was also noted that the gene expression patterns of cells from female patients clustered closely in PCA, except for patient 136. However, the reported sample size was insufficient to stratify CD8 T cell gene expression comparisons by sex, hence further investigation taking sex-effects into account is warranted.

Restoration of general CD8 T cell function after therapeutic resolution of chronic HCV could have beneficial effects on the function of antigen-specific cells and help prevent complications associated with chronic HCV and cirrhosis, including HCC and other immune dysfunction. Identification of the underlying mechanisms of widespread CD8 T cell hyperactivation in chronic HCV infection with AF may provide insight on targets for immune restoration following antiviral therapy. Additionally, targets of immune restoration identified in the context of chronic HCV may inform potential restoration approaches across other liver disease aetiologies including HBV or HIV co-infections and MAFLD.

## Data availability statement

The datasets presented in this study can be found in online repositories. The names of the repository/repositories and accession number(s) can be found below: https://www.ncbi.nlm.nih.gov/geo/query/acc.cgi?acc=GSE253673, GSE253673 (GEO).

## Ethics statement

The studies involving humans were approved by The Ottawa Health Science Network Research Ethics Board. The studies were conducted in accordance with the local legislation and institutional requirements. The participants provided their written informed consent to participate in this study.

## Author contributions

JL: Formal analysis, Investigation, Methodology, Software, Validation, Visualization, Writing – original draft, Writing – review & editing. AV: Data curation, Investigation, Validation, Writing – review & editing. DR: Investigation, Validation, Writing – review & editing. SPD: Formal analysis, Investigation, Resources, Writing – review & editing. WLS: Methodology, Resources, Writing – review & editing. CLC: Conceptualization, Methodology, Writing – review & editing. AMC: Conceptualization, Funding acquisition, Methodology, Project administration, Supervision, Writing – original draft, Writing – review & editing.

## Supplementary material

Supplementary Figure 1 CD8 T cells in untreated HCV-infected individuals express increased Hh signaling genes. Normalized relative mRNA expression of **(A)** *PTCH1*, **(B)** *SMO*, and **(C)** *GLI1* in isolated CD8 T cells from AF or MF patients, compared to cells from healthy controls (H), assessed by qPCR after 16h of stimulation using anti-CD3/CD28 antibodies. Multiple comparisons are analyzed by Kruskal-Wallis *H*-test with Dunn’s post-test **p ≤* 0.05.

Supplementary Figure 2 Gating strategy for isolated CD8 T cell function. Lymphocytes were gated for analysis, followed by two rounds of singlets gating to exclude cells clumps. Viable cells were gated using the Zombie Aqua viability dye prior to CD8^+^ cell gating. CD8 T cell function was assessed by the proportion of IFN-γ^+^ and perforin^+^ cells after 48h stimulation with anti-CD3/CD28 antibodies. T cell subsets were defined using markers CCR7 and CD45RA.

Supplementary Figure 3 Cell viability and dose response of cyclopamine-treated CD8 T cells. Isolated CD8 T cells from healthy donor blood were stimulated for 48h with anti-CD3/CD28 antibodies. **(A)** Cells remain viable with increasing doses of cyclopamine during stimulation. **(B)** IFN-γ and **(C)** perforin expression is ablated by cyclopamine in a dose-dependent manner. Multiple comparisons are analyzed by 1-way ANOVA with Dunnett’s post-test, **p ≤* 0.05, ***p ≤* 0.01.
