# Supplementary figures and images for: Lasting differential gene expression of circulating CD8 T cells in chronic HCV infection with cirrhosis identifies a role for Hedgehog signaling in cellular hyperfunction
Source: Front Immunol. 2024 Jun 3;15:1375485. doi: 10.3389/fimmu.2024.1375485 (PMC11180750; doi:10.3389/fimmu.2024.1375485)

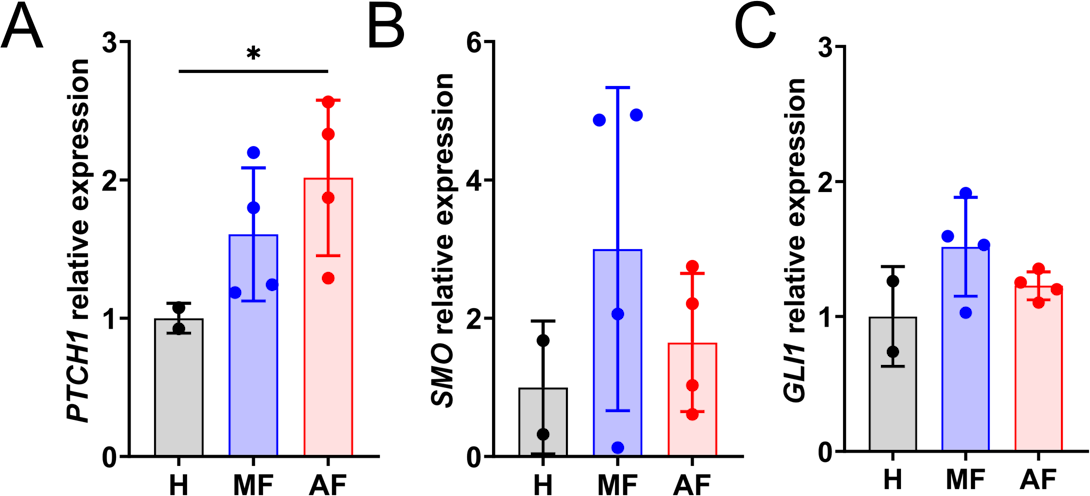

Supplement: Supplementary Figure 1 — CD8 T cells in untreated HCV-infected individuals express increased Hh signaling genes. Normalized relative mRNA expression of (A) PTCH1, (B) SMO, and (C) GLI1 in isolated CD8 T cells from AF or MF patients, compared to cells from healthy controls (H), assessed by qPCR after 16h of stimulation using anti-CD3/CD28 antibodies. Multiple comparisons are analyzed by Kruskal-Wallis H-test with Dunn’s post-test *p ≤ 0.05. [file Image_1.tif]

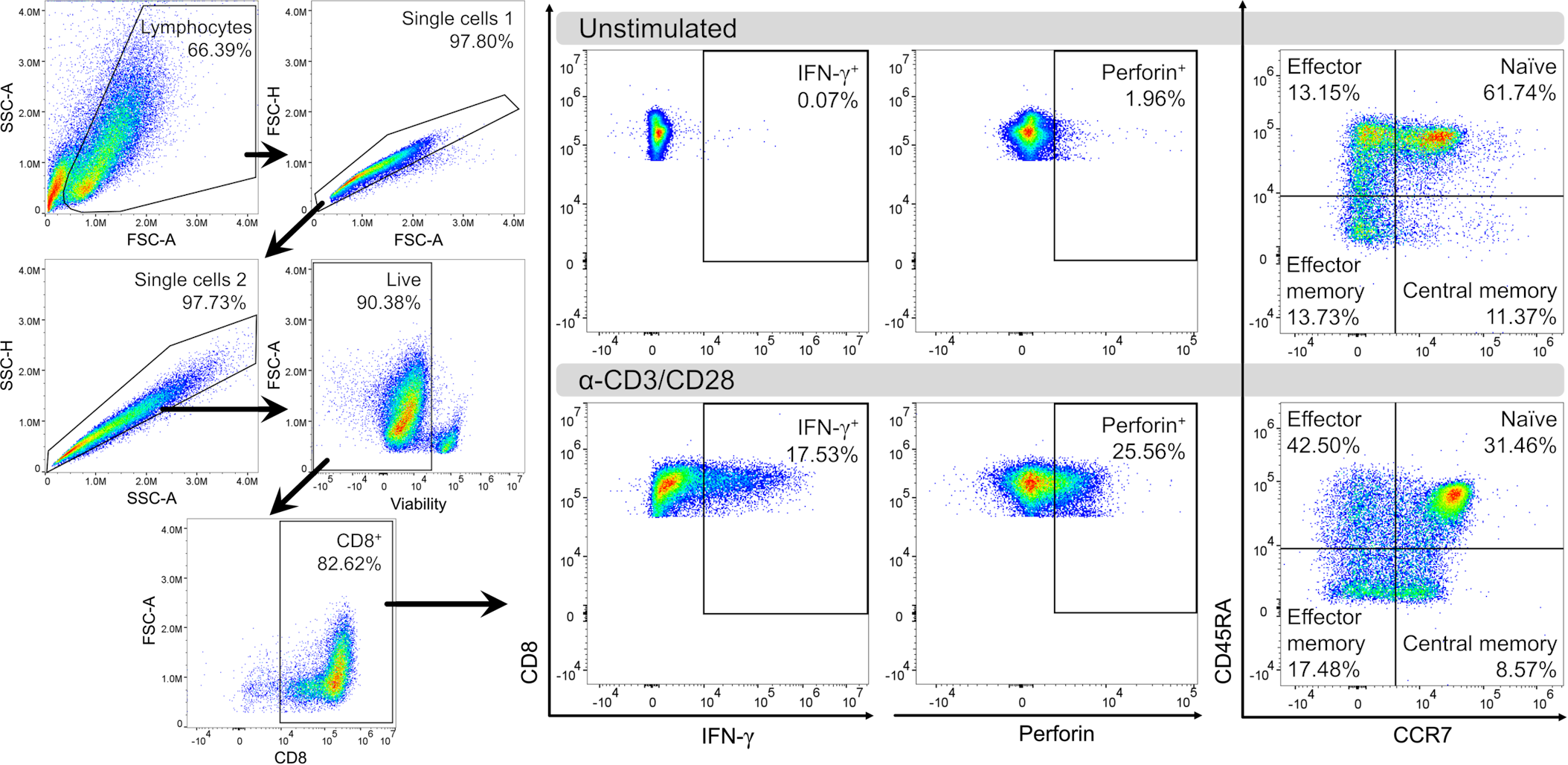

Supplement: Supplementary Figure 2 — Gating strategy for isolated CD8 T cell function. Lymphocytes were gated for analysis, followed by two rounds of singlets gating to exclude cells clumps. Viable cells were gated using the Zombie Aqua viability dye prior to CD8+ cell gating. CD8 T cell function was assessed by the proportion of IFN-γ+ and perforin+ cells after 48h stimulation with anti-CD3/CD28 antibodies. T cell subsets were defined using markers CCR7 and CD45RA. [file Image_2.tif]

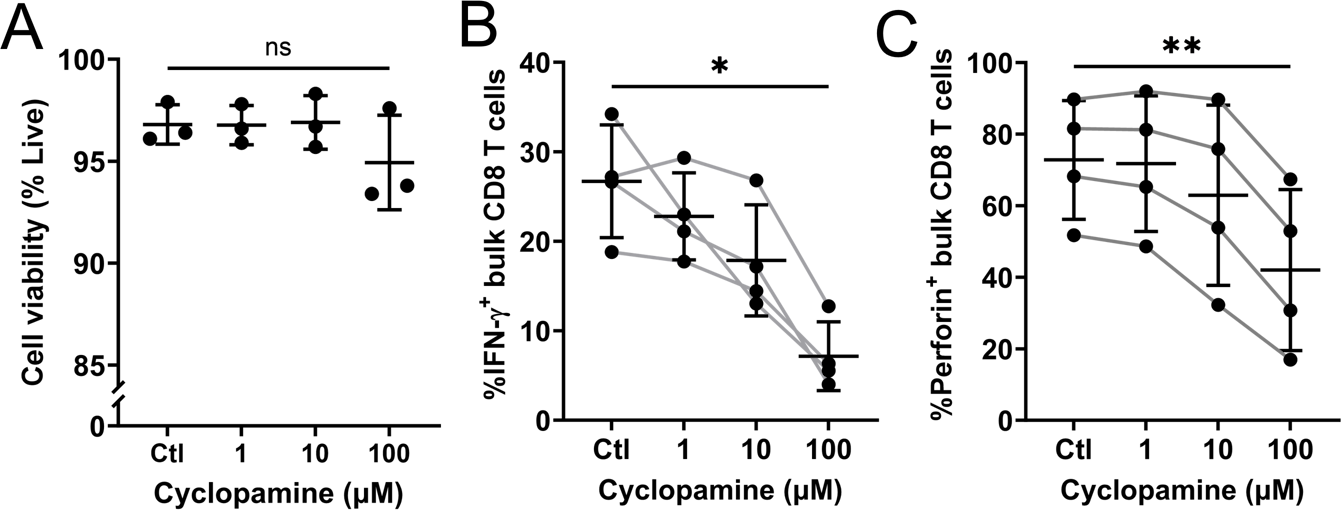

Supplement: Supplementary Figure 3 — Cell viability and dose response of cyclopamine-treated CD8 T cells. Isolated CD8 T cells from healthy donor blood were stimulated for 48h with anti-CD3/CD28 antibodies. (A) Cells remain viable with increasing doses of cyclopamine during stimulation. (B) IFN-γ and (C) perforin expression is ablated by cyclopamine in a dose-dependent manner. Multiple comparisons are analyzed by 1-way ANOVA with Dunnett’s post-test, *p ≤ 0.05, **p ≤ 0.01. [file Image_3.tif]
